# Functional Outcome of Solid Interlocking Nail in Open Tibial Fracture at a Tertiary Care Center: A Descriptive Cross-sectional Study

**DOI:** 10.31729/jnma.8571

**Published:** 2024-05-31

**Authors:** Vijayendra Adhikari, Prasamsha Sitaula, Ojas Thapa, Sumi Singh, Anil Kumar Mishra, Ramesh Prasad Singh, Pralhad Kumar Chalise, Praphulla Shrestha

**Affiliations:** 1 Department of Orthopaedics, Nepal Medical College and Teaching Hospital, Kathmandu, Nepal; 2 Nepal Medical College and Teaching Hospital, Kathmandu, Nepal; 3 Tribhuvan University Teaching Hospital, Kathmandu, Nepal; 4 Jacob's School of Medicine and Biomedical Sciences, University of New York, Buffalo, USA

**Keywords:** *open fracture*, *Gustilo-Anderson classification*, *solid interlocking nail*, *tibia*

## Abstract

**Introduction:**

There is a high incidence of open fractures accounting 23% of all tibial fractures. The minimal soft tissue and precarious blood supply of the shaft of tibia make these fractures vulnerable to complications. The treatment should be decided through thoughtful analysis for personality of injuries and the status of the soft tissue. Intramedullary nailing allows stable fixation with minimal soft tissues dissection and preserve the soft tissue and allows early joint motion with higher rate of union. The purpose of our study was to find the outcome of open tibial fractures lower than Gustilo type IIIb, that were treated by unreamed solid interlocking intramedullary nails.

**Methods:**

A descriptive cross-ectional study was conducted from December 2021 to June 2023 after taking approval from ethical committee. All 34 patients treated with solid interlocking intramedullary nail, without reaming for open tibial fracture during 18 months period were included in the study. Final follow up was done at one year and the outcome was assessed by Modified Ketenjian's criteria.

**Results:**

The mean time of union rate was 15.82±3.95 weeks. Complications were: superficial infections in 4 (11.76%) patients and deep infection in 1 (2.94%) patient. Using Modified Ketenjian's Criteria, 26 (76.47%) patients had an excellent result followed by good in 7 (20.59%), fair in 1 (2.94%) and there was no case with poor outcome.

**Conclusions:**

Solid intramedullary interlocking nail is an effective treatment with minimal soft tissue dissection for open tibia fracture less than GA III B as it provides stable fixation with early mobilization and provides a high rate of union, less complication and early return to function.

## INTRODUCTION

There is a high incidence of open fractures accounting 23% of all tibial fractures.^1^ The minimal soft tissue and precarious blood supply for shaft of the tibia make these fractures vulnerable to delayed union, malunion, nonunion and infection.^2^

External fixator and intramedullary nailing are common treatment options for the fixation of open tibial fractures.^2,3^ External fixator allows immediate stabilization with soft tissue care but is associated with significant rates of various complications like pin tract infection, malunion and conversion to definitive stabilization.^4^ Intramedullary nailing allows stable fixation preserves the soft tissue and allows early joint motion with a higher rate of union.^5^ The unreamed solid nails provide more stability, obliterate the dead space of nail and less chance of complications with high union rate.^6^

The purpose of our study was to find the outcome of open tibial fractures lower than Gustilo type III b, that were treated by unreamed solid interlocking intramedullary nails.

## METHODS

A descriptive cross-sectional study was conducted in the department of orthopedics of Nepal Medical College and Teaching Hospital after taking Ethical approval from the institutional review committee (Ref: 059-077/078). All the patients meeting the inclusion criteria during the study period were included in the study. A total of 34 patients with open tibial fractures were treated with solid interlocking intramedullary nails without reaming during a period of 18 months (December 2021 - June 2023).

Fractures were graded according to Gustilo-Anderson (GA) classification. Patients aged 18-50 years who presented with open tibial fractures were included. Gustilo type IIIB, type C open tibial fractures, Segmental fractures, communited fractures and polytrauma patients were excluded from the study.

Injectable antibiotics were started with broad-spectrum antibiotics followed by culture and sensitivity antibiotics until the 7^th^ post-operative day after which oral antibiotics were given. Extensive irrigation and debridement of the wound were done, followed by primary or delayed closure for Type I and II injuries. Severe open wound (GA II, GA III) was obtained by either skin graft or muscle flap later on.

Range of motion exercises were started from the first postoperative day with partial weight bearing followed by full weight bearing as pain tolerable.

Suture was removed after 2 weeks and all patients were followed up 6 weekly for the first 6 months and then every 3 months up to 1 year for evaluation of bony union which was assessed clinically by the absence of pain and tenderness at the fracture site and radiological assessment included the presence of a bridging callus with obliteration of the fracture line on both anteroposterior (AP) and lateral views.

Final follow-up was done at one year and the outcome was assessed by Modified Ketenjian's criteria and rated as excellent, good, fair or poor.^7^ Data was collected and analyzed using SPSS software.

## RESULTS

Of the 34 open tibial fractures included in this study, 7 (20.59%) patients were in the age group 18-26 years, 10 (29.41%) in the age group 27-34 years, 7 (20.59%) patients in the age group 35-42 years and 10 (29.41%) in the age group of 43-50 years. This study group included 10 (29.41%) females and 24 (70.59%) males with 10 (29.41%) patients having left-sided fractures and 24 (70.59%) having right-sided fractures. Among 34 patients, 24 (70.59%) of them were injured by road traffic accidents, 9 (26.47%) by fall injury and 1 (2.94%) patient by physical assault. In this study, the location of the fracture was in the middle third of the tibia in 27 (79.41%) patients, followed by a lower third in 5 (14.71%) and a proximal third in 2 (5.88%) of the cases. There were 26 (76.47%) patients with GA-I, 5 (14.71%) in GA-II and 3 (8.82%) in GA-IIIA. Among 34 patients, 32 (94.11%) fractures were reduced by closed technique and 2 (5.88%) fractures were reduced by the open technique and fixed with solid intramedullary nails with proximal and distal locking. The duration from injury to surgery in GA-I was 2.69±0.74 days, GA-II was 3.6±0.15 days and in GA-IIIA was 4±0.17 days.

The union rate in GA-I was 14.85±2.41 weeks, the GA-II union rate was 20±5.47 weeks and in GA-IIIA union rate was 22.67±5.78 weeks. The mean time of union rate was 15.82±3.95 weeks.

The complications observed in our study were superficial infections in four patients and deep infection in one patient. In patients with GA-I, there were no infections, in GA-II infection was present in three cases, and in GA-IIIA, infection was present in two cases with one deep infection (Table 1). The superficial infection was resolved by proper debridement and antibiotic therapy and debridement and exchange nailing was performed at 10 weeks for the deep infection case. Three patients all with GA-IIIA, where the secondary closure procedure required prolonged immobilization, suffered from knee stiffness. Delayed union was seen in one patient of GA-IIIA grade open fracture, eventually united after dynamization.

**Table 1:** Complications (n=34).

Complication	n (%)
Infection	5 (14.70)
Knee stiff	3 (8.82)
Delayed union	1 (2.94)

All the results were clinically evaluated using Modified Ketenjian's Criteria, 26 (76.47%) patients had an excellent result, good in 7 (20.59%) patients, fair in 1 (2.94%) case and no case with a poor outcome after one year of follow up (Table 2).

**Table 2:** Functional outcome of patients (n=34).

Outcome	6 weeks	12 weeks	24 weeks	1 year
Excellent	-	19 (55)	24 (70.59)	26 (76.47)
Good	-	9 (26.47)	7 (20.59)	7 (20.59)
Fair	16 (47.06)	5 (14.71)	3 (8.82)	1 (2.94)
Poor	18 (52.94)	1 (2.94)	-	-

## DISCUSSION

Open tibia fractures are commonly associated with soft tissue disruption that results in high complication rates including delayed union, malunion, nonunion and infections.^8^ So, the management of open fractures of the tibia is a challenging job.

Open tibial fractures are seen in the adult age group as they are the people engaged in various outdoor activities and as a result, most of them sustain highvelocity injuries. In our study, mean age of patients was 34.47±10.20 years comparable to Whittle et al mean age of 36 years and Khan I et al mean age was 33.28±13.83 years.^9,10^

In our study, the majority of the fractures were due to road traffic accidents: 24 (70.6%) this may be due to poor traffic sense and poor quality of roads, leading to a higher incidence of road traffic accidents (RTA) in our scenario while 9 patients (7.5%) were due to fall from height and physical assault in one patient (5%), which is comparable to a previous study done by Drosos GI et al (RTA - 29.8%, Fall from height - 29.25).^11^

There were 26 (76.5%) patients with GA-I, 5 (14.7%) with GA-II, and 3 (8.8%) with GA-IIIA. This is comparable to the study done by Seron S et al in which 16 fractures (22%) were classified as grade I, 38 fractures (51%) as grade II, and 20 fractures (27%) as Gustilo-Anderson grade IIIA open fractures.^12^

The mean time from injury to surgery in GA-I was 2.69±0.74 days, in GA-II, was 3.6±0.15 days, and in GA-IIIA was 4±0.17 days. Delayed presentation, infection and soft tissue injury were the cause of delayed surgery.

In this study, 32 (94%) fractures were reduced by closed technique and 2 (6%) fractures were reduced by open technique. Partial weight bearing was started immediately followed by full weight bearing as pain tolerable, with encouragement to achieve a full range of motion of the hip and knee of the affected limb.

The union rate in GA-I was 14.85±2.41 weeks, GA-II union was 20±5.47 weeks and GA-IIIA union was 22.67±5.78 weeks. The mean time of union was 15.82 ±3.95 weeks. In comparison, the study done by Agrawal A et al shows the average time to union was 16.0 weeks in grade I cases, 18.3 weeks in grade II cases and 23.6 weeks in grade III A cases and the mean time to union was 20.7 weeks.^13^

Altogether 5 cases ended up with an infection. In GA-I there were no infections, in GA-II infection was present in 3 (60%) cases and GA-IIIA infection was present in 2 (40%) cases. Kamat AS et al14 studied 12 out of the 103 cases of sustained infections, among them 1 was a Grade I fracture (8.3%), 3 were Grade II fractures (25%), and the remaining 8 were Grade III fractures (66.6%).^14^

Out of 34 patients, 5 patients had an infection, among them 4 patients had superficial infection that occurred at the site of a wound and it was healed with dressing andIV antibiotics but 1 patient had got a deep infection for which debridement and exchange nailing was performed at 10 weeks.

In this study, one case had delayed union, which was healed by 26 weeks. For this case, dynamization was done during 10 weeks follow-up. This was comparable with Salem KH et al who recommended dynamization within 10 weeks of surgery.^15^

The final assessment in our series was done using the Modified Ketenjian's Criteria, 26 (76.5%) have got excellent,^7^ (20.6%) have good, and 1 (2.9%) has a fair functional outcome. In comparison with the study of D Joshi et al, Agarwal A et al and Khairnar G et al showed better functional outcomes which may be due to stable fixation and early mobilization.^7,13,16^

## CONCLUSIONS

The unreamed solid intramedullary interlocking nail is an effective treatment with minimal soft tissue dissection for open tibia fracture lower than GA type III B as it provides stable fixation, an easier soft tissue coverage, early mobilization and provides a high rate of union, less complication and early return to function.
